# The detrimental effects of lead on human and animal health

**DOI:** 10.14202/vetworld.2016.660-671

**Published:** 2016-06-27

**Authors:** Mohammed Abdulrazzaq Assi, Mohd Noor Mohd Hezmee, Abd Wahid Haron, Mohd Yusof Mohd Sabri, Mohd Ali Rajion

**Affiliations:** 1 Department of Community Health, College of Health and Medical Techniques, Al_Furat Al_Awsat Technical University, Iraq; 2 Department of Veterinary Preclinical Sciences, Faculty of Veterinary Medicine, Universiti Putra Malaysia, 43400, Serdang, Selangor, Malaysia; 3 Department of Veterinary Clinical Studies, Faculty of Veterinary Medicine, Universiti Putra Malaysia, 43400, Serdang, Selangor, Malaysia; 4 Department of Veterinary Pathology and Microbiology, Faculty of Veterinary Medicine, Universiti Putra Malaysia, 43400, Serdang, Selangor, Malaysia

**Keywords:** antioxidant, free radical, lead poisoning, oxidative stress

## Abstract

Lead, a chemical element in the carbon group with symbol Pb (from Latin: Plumbum, meaning “the liquid silver”) and has an atomic number 82 in the periodic table. It was the first element that was characterized by its kind of toxicity. In animal systems, lead (Pb) has been incriminated in a wide spectrum of toxic effects and it is considered one of the persistent ubiquitous heavy metals. Being exposed to this metal could lead to the change of testicular functions in human beings as well as in the wildlife. The lead poising is a real threat to the public health, especially in the developing countries. Accordingly, great efforts on the part of the occupational and public health have been taken to curb the dangers of this metal. Hematopoietic, renal, reproductive, and central nervous system are among the parts of the human body and systems that are vulnerable toward the dangers following exposure to high level of Pb. In this review, we discussed the massive harmful impact that leads acetate toxicity has on the animals and the worrying fact that this harmful toxicant can be found quite easily in the environment and abundance. Highlighting its (Pb) effects on various organs in the biological systems, its economic, as well as scientific importance, with the view to educate the public/professionals who work in this area. In this study, we focus on the current studies and research related to lead toxicity in animals and also to a certain extent toward human as well.

## Introduction

Lead is considered as one of the most hazards and cumulative environmental pollutants that affect all biological systems through exposure to air, water, and food sources [1]. The exposure to lead induces clinic pathological changes through toxicity occurring in kidney and endocrine system [2]. A high level of lead in animals resulted in reproductive failure [3]. Lead is one of the global environmental pollutants, mainly found widely in industrial regions, as such that animals can easily be exposed to lead. Lead poisoning particularly in animals can be found from numerous sources in the general environment, and this can be traced back from contamination of feed, and soil from industrial pollution and agricultural practices. Furthermore, consuming high amount of lead has resulted in poor performance, poisoning, and death in animals [4,5]. Accumulated lead is toxic in most of its chemical forms, whether it is inhaled or ingested in water or feed. The extent to which orally administered lead absorbed into the host is small. However, due to its slow rate of elimination, harmful levels of lead can accumulate in tissues after prolonged exposure to low quantities [6]. Lead is a heavy metal that is both poisonous and a ubiquitous environmental toxicant. It is distributed in all parts of the environment in three main forms: Metallic lead, lead salts, and organic lead containing carbon [7,8]. The major sources of occupational lead exposure are traditionally leaded gasoline, lead-based paints, and batteries. Lead exposure has been a widely recognized as a significant public health problem over recent decades, and high levels of occupational lead contact are now strictly controlled due to their adverse health effects. However, environmental lead exposure such as that in domestic tap water, food-borne contamination, and household dust continue to pollute our surroundings in daily life [9]. Exposure to lead is considered to be detrimental and associated with behavioral abnormalities, hearing deficits, neuromuscular weakness, and impaired cognitive functions in humans and experimental animals [10]. Indeed, no blood-lead levels appeared to be safe, and sub-clinical influences of lead toxicity were reported in recent years [11]. This is attributed to the induction of oxidative stress by elevation of reactive oxygen species (ROS) like lipid peroxides, hydroxyl radicals and hydrogen peroxide and superoxide radicals [12]. In mammals, ROS is generated either by the mitochondrial electron transport chain or nicotinamide adenine dinucleotide phosphate oxidase in virtually all cell types and is involved in regulating cell proliferation and differentiation, and genomic stability [13]. However, increased ROS production occurs in numerous pathologic situations including hematopoietic stem cell (HSC) apoptosis and premature senescence. The oxidative stress role in the lead-exposure-induced HSC function remains unclear [14]. There are about 40 heavy metals which are capable of combining with a wide variety of organic molecules and are potent enzyme inhibitors because of their interaction with ligands present in proteins, and they inactivate the enzyme system of cell [15]. The removal of lead from gasoline has resulted in decreased exposure in many countries. However, because of its malleability, resistance to corrosion, and low melting point, lead has been widely used in industries and levels remain high in many areas [16].

The significance of the review is mainly due to the harmful effects of lead acetate toxicity has on the animals and its (Pb) abundance in the environment. Therefore, it is warranted for the review to be written to address this matter thus preventing more occurrence of lead toxicity and notably to highlight the effects lead toxicoses on animal husbandry economics and science.

The aim of this review paper is to educate the people who work closely with animals such as veterinarians, researchers, animal practitioners, and others regarding the detrimental effects of lead toward the health and well-being of the animal’s particularly food-producing animals. This elaborated review focuses on the current investigations related to lead toxicity.

### Lead Effects on Various Organs

Lead, which affects many cellular processes and enzyme systems all over the body, has different possible mechanisms of action. Most research on lead poisoning focuses more on its toxicity effects on the hematology, cardiovascular, renal, and neurotoxicities with the emphasis toward the neurological effects of lead poisoning. Yet, the other areas of concern were also plentiful from this research [17]. At a theoretical level, mechanisms of lead toxicity on the reproductive system of male were not yet fully explained. Numerous pathways are possible to clarify how exposure to lead could decrease fertility in male. For example, multiple potassium and calcium channel isoforms in human spermatozoa and testes might be contributed in the earlier activities of acrosome reaction [18]. Moreover, in lead-exposed rats’ reproductive organs, activities of some enzymes, such as sodium potassium ATPase and alkaline phosphatase (ALP), were shown to be decreased [19]. An additional issue in reproductive toxicity of lead may have a relation with the excessive generation of ROS, an issue that was recently given much attention. Sulfhydryl antioxidants production is inhibited by ROS, which damages nucleic acids, inhibits the repair of DNA and inhibits reactions of enzyme, and also inhibits the peroxidation of initiating lipid in cellular membranes. Oxidative stress is induced by lead, which encourages the hydrogen peroxide generation [20]. The negative wide-ranging of effects, because of a raise in the levels of ROS in tissues, were assumed as a major disorders contributor which relates to the exposure to lead [21]. An epidemiological study of the male reproductive system has demonstrated positive correlations between seminal plasma lead and spermatozoa ROS levels [22]. Alternatively, increased activity of superoxide dismutase (SOD) was observed in people with prolonged lead exposure and it proposes a mechanism to the increasing of ROS production which is caused by exposure to lead [23]. This might be resulted in oxidative cell in reproductive tissues damage which is closely related to the production of ROS. For instance, research on the sperm of rats under ROS exposure *in vitro* decreased the rate of penetration in the zona-intact ova and showed an early acrosome reaction [24]. Nevertheless, lead-induced oxidative stress has different responses from low to high doses in numerous target sites, in addition to sperm [25]. Further evidence of animals that have been chronically exposed to lead was a rise on lipid peroxide concentration in the reproductive organs [26]. Therefore, some studies have proposed that increased ROS production induced by lead is a significant molecular mechanism for the disorder in reproduction in male during spermatogenesis or in the hormonal stages. ROS are the by-products of many degenerative reactions in many tissues. They will affect the regular metabolism and damage the cellular components, because this molecule has one or more unpaired electrons, making it highly reactive with other molecules. ROS can damage cell structures of those of carbohydrates, nucleic acids, lipids, and proteins and alter their functions and destroy the living cells. The shift in the balance between oxidants and antioxidants in favor of oxidants was termed “oxidative stress” [27]. The ability of lead for the production of ROS result in DNA strand breaks and replace zinc in DNA binding proteins [28]. Accordingly, interest has recently grown in the role and usage of natural antioxidants like fruits and vegetables as a strategy to prevent oxidative damage in various health disorders with oxidative stress [29]. The toxicity effects of lead acetate are confirmed by previous study of Elgawish and Abdelrazek [30] that suggested SOD was reduced in lead acetate-treated rats compared to the other groups. The lead is reported to cause oxidative stress by generating the release of ROS such as superoxide radicals, hydrogen peroxide and hydroxyl radicals, and lipid peroxides. There has been increased interest among phytotherapists to use medicinal plants with antioxidant activity for protection against heavy metal toxicity [31].

Lead intervenes with the endogenous development of opiate system [32]. Many toxic properties of lead are due to its ability to mimic or compete with calcium. At picomolar concentrations, lead competes successfully with calcium for binding sites on the cerebellar phosphokinase C and thereby affects neuronal signaling [33]. Lead has a binary impact on neurotransmitter release: Spontaneous neurotransmitter release is enhanced, whereas stimulated release is inhibited [34]. The synthetic pathway of the heme is also considered as one of the target sites for lead toxicity. Delta aminolevulinic acid dehydratase (ALAD) has a high sensitivity to lead. Yet, the inhibition of this enzyme seems to be found in the increasing in circulating ALA, which is known as a weak gamma-aminobutyric acid (GABA) agonist which reduces the releasing of GABA via presynaptic inhibition. Increased in circulating ALA might be the reason behind the observation of some disorders in behavior noticed in patients with porphyria and maybe in toxicity of lead. Behavioral alterations which are secondary to exposure to lead in primates and rodents are similar to those in human. A study revealed monkeys that consumed food containing lead acetate from birth to 200 days of age have reached level from 3 to 25 ¹g/dl of lead in blood. At the age from 7 to 8 years, they have been given a test of late alternation, in which the critical positive stimulus was alternated, and hence, treated monkeys exhibited less learning ability, mainly at longer delay intervals [35]. In the population, contact to lead arises mainly through the oral route, while the industrial contact is mainly through inhalation. Lead has the same toxicity unrelated of access method into the body; therefore, the toxicological data would not depend on the route of exposure [17]. According to many laboratory experiments on animals, the lead has an extensive range of toxic influences through different systems of an organ. However, lead may influence the gastrointestinal (GI), cardiovascular, immune, hemolymphatic, reproductive, urinary, and nervous systems and also cause tumors in laboratory animals. The most significant studies relating to the level of lead produced in the blood to the oral dose. In male rats, Chowdhury *et al*. [36] detected that the sperm counts, with levels of lead of 72 µg/dL in blood, were reduced. However, there were no significant effects in male rats, which had levels of 54 µg/dL of lead in blood. In female rats, which had levels of 30 µg/dL of lead in blood, they have unbalanced estrus cycles. Ovarian follicular cysts have been resulted in female rats that had lead levels of 53 µg/dL in blood. In male rats that had levels of 19 µg/dL of lead in blood, the prostate weight was significantly increased. In addition, a testicular damage in male rats which had levels of 30 µg/dL of lead in blood was detected. Davidovics and DiCicco-Bloom [37] stated an *in vitro* changes valuation to mammalian neurogenesis via a model of well-characterized cortical precursor following exposure to lead. In rat’s kidney, an influence of lead on lipid peroxidation, renal function, and expression of heme oxidation was evaluated by Vargas *et al*. [38]. As stated by Qian *et al*. [39], the production of glucose-regulated protein 78 (GRP78) in a protecting response to lead was raised. Researchers presented cultured C6 glioma cells in rat, a cell line of astroglia-like, to lead acetate of 1 µ for a week. They found that the intracellular levels of two proteins were increased; of which, one has been GRP78. Regarding the GRP78, an accumulation started within 24 h and proceeded during the exposure time. Free radical-induced damage caused by Pb and Cd is accomplished by two independent but related mechanisms [40]. Another mechanism for Pb- and Cd-induced oxidative stress is their effect on the antioxidative defense system of cells. Pb and Cd have a high affinity for —SH groups in enzymes of the antioxidative defense system such as glucose-6-phosphate dehydrogenase (G6PD), glutathione (GSH) peroxidase (GPx), catalase (CAT) and SOD, and subsequently, inhibit their activity [40-42]. In all rats treated with lead, no major differences were observed in the absolute liver weights. Alternatively, the comparative liver weights have been considerably raised in animals which were exposed to the highest concentration of PbAc (p<0.05) comparing to the control group. Lead toxicity caused a significant increase in serum alanine aminotransferase (ALT), aspartate aminotransferase (AST) activities, and a significant decrease in total serum protein as compared to their corresponding controls [43].

### Lead Effects on Cardiovascular System

For cardiovascular changes, important neural and hormonal systems were affected by lead. These systems donate to the rule of the resistance of peripheral vascular, cardiac output, and heart rate [44]. Vascular smooth muscle might be directly affected by lead via constraining the activity of Na-K-ATPase, with a related raise of the levels of intracellular calcium [45]. Lead was also found to affect blood pressure. Bagchi and Preuss [46] stated that the systemic blood pressure in young Sprague-Dawley rats have changed and bone mineral density have decreased after exposure to 1% of lead acetate for 40 days in the drinking water. After treatment continued, systemic levels of blood pressure were raised high and eventually returned to normal. However, after several months of lead exposure ends, it rises again above the accepted levels. In rats, lead-induced hypertension was related to nitric oxide reduction that is involved in (1) down-regulation of the soluble enzyme guanylate cyclase which gives cyclic guanosine monophosphate (a nitric oxide-induced vasodilation mediator); (2) regulating blood pressure; (3) adrenergic system changes (i.e., raised in plasma norepinephrine, reduced the density of vascular β-adrenergic receptor, and raised the activity of central sympathetic nervous system) [17].

Acute and chronic lead poisoning contributes in vascular and cardiac damage as well as possible fatal consequences such as cardiovascular illnesses and hypertension [47]. Exposure to low level of lead may cause hypertension in both humans and animals [48]. Cerebrovascular accidents, peripheral vascular disease and ischemic coronary heart disease may also be considered as major disorders. Although indication of causative relationship of hypertension and exposure to lead was specified, it is only applied in cardiovascular outcomes cases of toxicity of lead [47]. In animals, effects of cardiovascular following lead exposure were recently studied by Vaziri and Sica [44]. Kilikdar *et al*. [49] showed that the rats when receiving (15 mg/kg BW) of lead acetate cause heart damage was clearly evident from increased activities of serum glutamate oxaloacetate transaminase and lactate dehydrogenase.

### Lead Effects on Kidney and Liver

Exposure by the high level of lead (>60 µg/dL) may cause renal dysfunction. Even a low level of lead (~10 µg/dL) may also provide the same problem [50]. There are two types of abnormality in renal functionality: Acute and chronic nephropathy. Acute nephropathy can be categorized morphologically via the degenerative changes present in the tubular epithelium accompanied by the presence of nuclear enclosure bodies, which contain lead protein complexes, and functionally via a mechanism of impaired tubular transport. Yet, it is not the reason behind the appearance of protein in the urine but it could increase a nonstandard secretion of amino acids, phosphates, and glucose, a mixture mentioned to as Fanconi’s syndrome. However, chronic nephropathy is much simpler and may cause irreversible morphological and functional changes. However, it is categorized by tubulointerstitial and glomerular variations, resulting in hyperuricemia, hypertension and renal breakdown [51]. Carmignani *et al*. [52] stated in his review that the pathological influence of the exposure of lead on the renal systems of both men and animals seems to result to the renal toxicity development under the influence of the oxidative stress that it causes. However, the previous study suggested that such effect only damages kidney in chronic exposure that becomes clinically significant, and that kidney damage does not usually occur in asymptomatic/acute cases.

Literature data on the effects of acute Pb exposure triggering oxidative stress in the kidney of animals are rare. The administration of lead acetate showed a significant (p<0.05) increase in urea and creatinine concentration compared with other groups [53]. Sharma and Singh [54] have recently reported that 24 h exposure to 10 and 150 mg/kg BW of Pb acetate induced increased in renal thiobarbituric acid reactive substances (TBARS) content that acts as an indicator of lipid peroxidation, as well as SOD and CAT activities in kidney of Balb-c mice. Several authors have also confirmed that there were increased in lipid peroxidation in the kidneys of Pb-exposed animals. Studies have indicated that intraperitoneal (IP) injection of 20 mg/kg BW of Pb-acetate for 5 days and IP administration of 5 mg/kg BW of Pb acetate for a period of 30-day could induce increased in renal lipid peroxidation [55,56]. These findings are in agreement with Sharma *et al*. [57] who reported a significant increase in the level of renal TBARS in rats treated orally for 40 days with Pb-nitrate at a dose of 50 mg/kg BW as well as with the results of Wang *et al*. [16] who demonstrated lipid peroxidation enhancement as measured by the levels of malondialdehyde (MDA) in the kidney of rats exposed to 500 mg Pb/L via drinking water for 8 weeks. Recent investigations performed on mice [54,58] also showed that prolonged Pb exposure induced generation of free radicals and lipid peroxidation in the kidney, subsequent loss of membrane integrity and inactivation of tubular cell constituents. Most of the authors agreed that Pb produced lipid peroxidation mainly via ROS generation such as H_2_O_2_ and OH, although experimental data indicate the involvement of nitrogen species as well. This is supported by a study that showed IP injection of Pb-acetate (20 mg/kg BW) for 5 days induced an elevation of renal NO as a result of increased activity of inducible NO synthase [55]. Enhanced levels of ROS can also be attributed to Pb-induced disruptions in the antioxidant defense system. Several studies have shown that Pb alters the activity of antioxidant enzymes such as SOD, CAT, GPx, GST, and G6PD as well as the content of GSH in animals and humans. Which means that prolonged IP or oral Pb exposure in experimental studies induced significant decrease in the activity of antioxidant enzymes in renal tissues [16,55,56]. Mechanisms of Pb effects on these enzymes can be complex, since Pb can competitively inhibit bio-elements absorption and/or replace them in the active sites of enzymes or bind to — SH groups of proteins [16,56]. In addition to oxidative stress as mechanism of Pb toxicity in the kidney, the experimental studies have shown that Pb exposure induces an increase in apoptosis not only in liver but also in kidneys as well [59]. Has demonstrated that chronic treatment of rats with Pb-acetate over a period of 12-week increased the number of apoptotic bodies in the proximal tubular cells. Thus, it can be hypothesized that Pb intoxication influences gene expression of apoptosis proteins. After absorption, lead is passed to many tissues in the body. It causes basophilic stippling in blood because of its inhibitory influence on the enzyme α-amino levulinate dehydratase that influenced heme synthesis [60]. Renal toxicity arises in two ways, either by reversible commonly discovered after serious exposure of lead acetate in children or by irreversible interstitial nephropathy usually noticed during long-term exposure to lead [60]. Histopathological changes in the renal proximal tubular epithelium were derived from lead toxicity, and these changes cause interstitial nephritis and usually related to hypertension. It accumulated in proximal involuted tubules of renal cortex that produce both biochemical and morphological evidence of lead toxicity. An extensive work to study the severe effects of lead toxicity toward the kidneys has been well-documented [61]. Another trigger for lead-induced renal toxicity is from the exposure toward environmental lead poisoning and fast becoming an increased health burden to both animals and humans [62]. In this exposure, an acute lead-induced renal damage can occur in the absence of acute intoxication so that occult lead nephropathy may not be recognized as such [62]. While in chronic accumulation of lead in the body eventually leads to impairment in renal function [63]. Urea and creatinine are a waste product of amino acid metabolism they removed by kidney [64]. Oxidative stress appears to be involved in the development of renal toxicity induced by the environmental lead exposure that causes significant pathological lesions on the renal systems of men and animals [65]. Conterato *et al*. [66] investigated blood levels of Pb and Cd and parameters of oxidative stress in battery-manufacturing workers, painters from an automotive industry and workers who were not occupationally exposed to these metals, who served as controls. Although MDA levels were not increased in the exposed groups, elevated activity of GST was observed in both battery-manufacturing workers and painters. The correlation analyses revealed that Pb and Cd contribute to the elevation of this enzyme activity that was explained by the ability of these metals to induce GST gene expression. On the other hand, the increased GPx and SOD activities, observed only in the battery manufacturing workers, correlates with the increased of Pb blood levels, while CAT activity, changed only in painters, inversely correlated to Cd blood levels. These changes suggest an additive effect of Cd and Pb on blood GST levels, while other alterations in oxidative stress parameters were distinctively associated with either Pb or Cd blood levels. Hence, there may be a lack of interaction between Pb and Cd on the level of oxidative status in blood after coexposure by inhalation. Another investigation with the results supporting the possible antagonism between Pb and Cd was carried out on bank voles, animals that served as a good model for environmental lead exposure. Animals were exposed for 6 weeks of dietary Cd and Pb (Cd-60; Pb-300; Cd-60 + Pb-300 µg/g dry weight-concentrations two-fold higher than those expected in high contaminated areas). No significant changes in the liver concentrations of toxic metals, TBARS or GSH levels were observed between groups treated with a single metal and a group treated with a mixture [67,68] administered to rats by gavage for 90 days, three different doses, in an equitoxic mixture ratio, where the highest dose was 1/10 of the previously established LD50 for a mixture of Pb nitrate and Cd-chloride. Results indicated a strong, significant increase in MDA and decrease in GSH content in the liver and kidneys with decreased activities of antioxidant enzymes SOD, CAT, GPx. However, this experimental study did not give the information on the individual effects of Cd or Pb treatment under the same experimental conditions, and therefore, it is difficult to discuss possible interactions between these metals. In the same experiment, MT-1 and MT-2 gene expressions were investigated in rat liver and renal cortex and their significant up-regulation in both liver and kidneys were shown. The activation of defense systems was explained by the antioxidant effect of MT, availability of cysteine in MT for GSH synthesis, and by MT mRNA transcriptional response triggered by ROS. They also speculated that these changes could be related to the inhibition of steroid organic enzyme activity and effects on testosterone levels, sperm count and motility after coexposure to Pb and Cd. Literature data indicate that, similarly to Cd or Pb exposure, coexposure to these toxic metals induces apoptosis. In a 90-day subchronic oral toxicity study, relatively low and environmentally realistic concentrations of lead and cadmium induced significant hepatic and renal apoptosis that consequently impaired their function [69]. Pb hepatotoxicity has been related to the elevation in the levels of serum liver enzymes AST, ALT, and ALP [70] and alterations in hepatic cholesterol metabolism [71]. Due to its specific role in the organism, the liver is, besides blood, the main target of Pb-induced oxidative stress. Acute IP administration of 15 mg Pb-acetate/kg BW for 7 days [72] and 25 mg Pb-acetate/kg BW for 5 days [71] induced decreased in SOD and CAT activity and a two-fold decrease in GPx activity. These changes were followed by a significant decrease in the levels of GSH, partly as a result of biliary excretion of Pb bound to the SH groups of GSH, as proposed by Abdou and Hassan [71]. Investigation of the recovery from Pb-induced oxidative stress in the rat liver was undertaken by Omobowale *et al*. [70]. Animals were given 0.25, 0.5, and 1.0 mg Pb-acetate/mL for 6 weeks and then were not treated for another 6 weeks. Increase in liver function test parameters, ALT, AST, ALP, as well as MDA and H_2_O_2_ concentrations in liver, and decrease in antioxidant enzymes SOD, CAT, GPx, GST activity and the content of GSH were observed after 6 weeks of Pb treatment. At the end of the experiment, only SOD activity was recovered and was within the accepted range, pointing to incomplete recovery from the effects of Pb-induced oxidative stress on other enzymes. The most important impact of Pb-induced oxidative stress in the liver is lipid peroxidation [70] that causes the alteration of membrane integrity and fatty acid composition and is associated with the increase of MDA level in the liver [73]. In another experiment, with the objective to investigate the gender, dose and time-dependent manner of Pb-induced oxidative stress besides observing time and dose-dependent increase in liver MDA levels; the correlation between oxidative stress, apoptosis, and mitogen-activated protein kinases (MAPKs) in hepatocytes was observed. The authors concluded that apoptosis might be induced via oxidative stress-mediated alterations in MAPKs [74]. Kilikdar *et al*. [75] stated that when the rats received lead acetate at a dose of 15 mg/kg BW IP and showed significantly increased in ALP activity by 62% in comparison to the activity measured in control animals, and rats when treated with lead acetate for 7 consecutive days. From the same study, a significant increase in the level of serum bilirubin by 38% in comparison to the values obtained in the control rats was also documented. There was no significant difference when compared to the control group in serum activities of LDH, ALP, ALT, AST while glycemia was slightly higher in rats exposed to 0.3% PbAc, but albumin and levels of TP were lower in all groups treated with lead [76].

Mice injected IP with lead acetate at a dose of 10 mg/kg showed a mild significant elevation of serum ALT only after 24 h (p<0.05) while at 100 mg/kg, it causes a significant time-dependent increases in serum ALT (p<0.001), when compared to the control group [77].

### Lead Effects on Neurobehavioral

The neurotoxicity of lead at high level of exposure has been well-documented for both man and animal species. Both the peripheral and the central nervous system are influenced by lead exposure. In children, the central nervous system is more influenced whereas, in adults, it is more on the peripheral nervous system [78]. Encephalopathy (an advanced degeneration of some parts of the brain) is a condition manifested significantly when comes into contact with lead, and the main symptoms include hallucinations, irritability, poor attention span, dullness, memory loss, muscular tremor, and headache. At high exposures, much simpler appearances arised and this includes paralysis, delirium, coma, convulsions, ataxia, and lack of coordination [79]. However, young children as well as unborn young are particularly vulnerable to the neurological influences cause by lead intoxication as the developed nervous system absorbs more portion of lead. Compared to adults, the amount of lead which is systemically circulating having contact to the children brain is meaningfully advanced [80]. Children might be hyperactive, easily irritated, and non-attentive even at the low level of exposure to lead. Nevertheless, children with higher levels of lead intoxication might have decreased intelligence, delayed growth, hearing loss and possessing only short-term memory ability. Permanent brain damage and even death might be a result of higher exposure to lead [81]. Some evidence is signifying that IQs with attentiveness, behavior of the child and concentration ability are influenced the low level of lead exposure significantly. An impact of exposure of lead to the peripheral nervous system was detected in a form of peripheral neuropathy, including decreased motor activity due to loss of myelin sheath that isolates the nerves, therefore totally damaging the nerve impulses transduction, resulting in deficiency of muscular coordination, fatigue and muscular weakness, particularly of the exterior muscles [82]. As stated by Mustafa and Hussein [83], administration of lead acetate to four rats from the group that showed many negative neurological signals, however, such as a decrease in vitality; muscle-mass weakness; tremors, along with lack of stability and equilibrium; an abnormal gait. One critical event during astrocyte differentiation is the change in the expression of the glial marker called glial fibrillary acidic protein [84]. Lead acetate can be found stored in the cerebellum, disturbing its physiology as well as causing neurotoxicity, cellular deterioration, and possibly cellular death [85].

### Lead Effects on the Hematopoietic System

Hematological influences were verified in animals and humans after lead exposure. These influences contain urinary porphyrins raised levels, δ-ALA, coproporphyrins, erythrocyte protoporphyrin, and zinc protoporphyrin. The results are the outcome of the three enzymes alteration, which involved in the biosynthesis of heme: δ-aminolevulinic dehydrase, ferrochelatase, and δ-ALA synthetase (ALAS) [17]. The hematopoietic system would be directly affected by lead by restraining the hemoglobin (Hb) synthesis through prevention of several key enzymes, which are parts of the pathway of heme synthesis. Yet, it decreases the circulating erythrocytes life span via raising the cell membranes fragility. Hence, anemia would be the result of the joint outcome of these two processes [51]. Lead has toxic effects in a wide variety of organs, causing impairments in the nervous, hematopoietic, renal, cardiovascular and reproductive systems following ingestion, inhalation or skin absorption [51]. There are two types of anemia resulted from lead poisoning: Frank anemia, that only occurred when the level of lead in blood is significantly raised for an extended period of time and hemolytic anemia, that is related to a seriously high level of exposure to lead [86]. The heme synthesis pathway suggestively affected by lead in a manner of dose-dependent via three main enzymes down-regulating that is included in the synthesis of the heme; the mitochondrial enzyme ferrochelatase that catalyzed the insertion of iron to protoporphyrin in order for the heme to be formed, δ-ALAD, the cytosolic enzyme by which the formation of porphobilinogen from δ-ALA is catalyzed, and finally, ALAS, a mitochondrial enzyme by which the formation of ALA is catalyzed [87].

Oxidative stress was understandably the major mechanism of lead toxicity. One of the main effects of lead toxicity in the hematopoietic system involves impairment of the Hb synthesis pathway through disrupted expression of genes encoding δ-ALAD, ferrochelatase, and ALAS [87]. In the mitochondria, the final steps, as well as the initial steps, of the synthesis of heme occur, while the intermediate steps occur in the cytoplasm. Yet, the three vital enzymes of this pathway, which have been mentioned above, inhibited by lead but confined only for clinical use to measure the degree of lead poisoning and its influence on ALAD is higher. Accumulation of ALA leads to restriction of ALAD, noticeable in the urine and plasma even at level of <10 µg/dl of lead in blood. While ALAD inhibition is known earlier at 10-20 µg/dl of lead levels in blood, biosynthesis of heme does not reduce till the action of ALAD is restricted by 80-90% that takes place at a higher concentration of lead of about 55 µg/dl in blood [88].

Ferrochelatase inhibition resulted in accumulation of protoporphyrin in erythrocytes and raised secretion of coproporphyrin in urine. Furthermore, restriction of this enzyme resulted when iron is substituted by zinc in the ring of porphyrin to form zinc protoporphyrin (ZPP). ZPP concentration therefore rose, and this could be used as a signal for monitoring the level of exposure by lead [89]. Hence, the production of heme is blocked by the cooperative restriction of those three key enzymes by the synthesis pathway of heme. The responsible mechanism for the shortening of erythrocytes life cycle was not established well. However, one of the most basic hematological influences observed from lead poisoning showed basophilic stippling’s of red cells in blood (existence of dense material in red cells of blood), that can be a possible biomarker to established poisoning by lead. Those collections are the products of ribonucleic acid degradation [21]. Kilikdar *et al*. [75] stated when rats were treated with LA IP for 7 consecutive days; the Hb content of blood significantly decreased by 25% in comparison to the value observed in the control rats. Erythrocytes are considered as the most vulnerable cells toward oxidative stress from lead as they have very limited reservoirs of antioxidant enzymes to counter the effect of ROS. In addition, they are unable to replenish antioxidant enzymes because they lack rough endoplasmic reticulum and becoming much prone to the damage by ROS. A number of red cells are used to indicate anemia produced by lead intoxication [90]. Lead toxicity also contributes toward the reduction of total Hb, red blood cells (RBCs) count and also the plasma level of T3 and T4 without significant changes for white blood cells (WBCs) count [91]. This finding was supported by Ibrahim *et al*. [92] in which it states that lead acetate when given orally to female rats at a dose of 10 mg/kg BW revealed significant decrease in Hb concentration, mean corpuscular Hb concentration, RBC count, and packed cell volume, whereas showed significantly increase the percentage of monocyte and mean corpuscular volume, total proteins, and WBC count.

### Lead Effect on Bone

In the human body, bones are the primary location for the storage of lead [93]. Lead is supposed to be stored in two sections in the bones. The non-exchangeable pool present deep in the bone cortex and the exchangeable pool located at the bone surface. As lead may get access to plasma easily from the exchangeable pool, it is actively being re-absorbed, it could transfer to the surface after leaving the non-exchangeable pool [21]. In adults, isotope procedure for stable lead revealed that bones contribute nearly 40-70% of the discharged lead in the blood. Around 85-95% of lead is stored in adults’ bones, while in children; about 70% of lead in a high concentration was detected in soft tissues. Lead mobilization and storage in bones rest on a number of factors such as age, dose/rate of pregnancy, exposure to lead, race, and gestation. Al Naimi *et al*. [94] stated that administration of lead acetate at 75 mg/kg BW at 20 and 40 days cause moderate hyperplasia of hemopoietic tissue with proliferation of megakaryocytes and the presence of thin trabeculae of calcified cartilage covered by a thin layer of bone. The bars of mineralized cartilage which resulted from impaired resorption of osteoclasts are wide and project further into the metaphyseal marrow cavity as compared to normal healthy bones.

### Lead Effect on Reproductive System

Elgawish and Abdelrazek [30] stated that lead acetate caused a significant decrease in functions of male reproductive organs as well as the occurrence of testicular tissue alterations in the histological patterns of testis. Samples of semen and blood have been analyzed from workers of factory manufacturing batteries and showing an opposite association between sperm concentration and volume and lead level in plasma. Important associations have been observed between the levels of protoporphyrin, dehydratase, and lead and reproductive parameters, exhibiting a reduction in the motility and density of sperm and viability counts and increased in the morphology of abnormal sperm head [95]. In both women and men, adverse effects on the reproductive system are due to the effect of lead. General effects found in men include: Abnormal spermatogenesis (decreased number and motility), reduced libido, abnormal prostatic function, chromosomal damage, changes in serum testosterone and infertility. In women, lead toxicity is more subjected to miscarriage, pregnancy hypertension, infertility, premature membrane rupture, premature delivery and pre-eclampsia [12]. Furthermore, direct effect of lead on the progressive stages of the fetus was also studied during the gestation period [96]. Experimental findings revealed that animals exposed to lead exhibited lower levels of plasma luteinizing hormone (LH) after stimulation with gonadotropin-releasing hormone as compared to controls as well as reduction in inhibin/follicle-stimulating hormone (FSH) ratio [97]. The rats treated with lead at a dose of 30 mg/kg BW presented considerable decrease in T3, T4 and TSH levels in serum, and histopathological findings exhibited an enlarged thyroid follicles associated with flattened epithelium as compared with negative control animals [98]. Saleh *et al*. [99] stated that there is an important reduction in the mating index and fertility index in females that were mated with lead-exposed male. Lead-exposed rats managed with testosterone took a less start-time to impregnate females. Marginal rise in number of implantations and decrease in pre- and post-implantation loss in females mated with rats coadministered with lead and testosterone indicates increased in fertility efficiency. Nevertheless, no important improvement in fertility was detected in normal rats injected with testosterone alone comparing to that of control rats [99]. Meanwhile, lead acetate administered orally to adult male rats at exposure of 273 or 819 mg/L cause significant decrease in the weight of the reproductive organs, reduction in epididymal sperm count, motile sperm, and viable sperm indicating a significant decreased in sperm production and deteriorated sperm quality and in serum testosterone levels in treated rats indicating a decreased steroidogenesis [100]. Lead acetate administered orally to rats at a dose of 8 mg/kg BW showed a significant decrease (p<0.05) in the reproductive organs and some accessory gland and also showed effect on levels of LH and testosterone hormone and sperm count accompanied by an increase (p<0.05) in mitotic index and percentage of abnormal sperm [101]. Hammed *et al*. [102] stated the exposure to lead acetate to rats orally at dose of 10 mg/kg BW showed a significant decrease effects in the level of LH and FSH when compared to the control group.

### Molecular Mechanisms of Lead Toxicity

Oxidative stress which plays an important role in lead poisoning is caused by an imbalance in the production of free radicals and compromising the system’s ability to readily detoxify the body of those radials resulted in cellular damage [12]. It starts with the onset of oxidative stress that has been proposed occurring in two simultaneous pathways; first, the generation of ROS such as hydro peroxides (HO_2_^•^), singlet oxygen, and hydrogen peroxide (H_2_O_2_). Second, the depletion of natural-occurring-antioxidants reserves [103]. The body’s antioxidant enzymatic system works to neutralize the ROS generation, with the cell’s most important enzyme which is the GSH. GSH is a tripeptide (containing sulfhydryl groups) found in mammalian tissues which destroy free radicals [104]. GSH exists in two forms; reduced (GSH) and oxidized GSH dismutase (GSSG). In reduced state, GSH donates reducing equivalents (H^+^ + e^–^) from its thiol groups in cysteine residues to ROS, thus making them stable. After donating its electron, it then readily combines with another molecule of its own to the form GSH disulfide (GSSG) in the presence of GPx. The reverse/opposite reaction generates GSH GSSG in the presence of GSH reductase (GR). Normally, only about 10% of GSH exists in the oxidized form (GSSG), the rest exist in reduced form as GSH. However, GSSG concentration is much higher than that of GSH during oxidative stress.

Lead shows a strong electron sharing property which aids in the formation of covalent bonds. These covalent bonds are formed between the lead moiety and the sulfhydryl groups in the antioxidant enzymes, making the enzymes most susceptible targets for lead, eventually rendering them inactive. On the other hand, lead inactivates GSH by binding with its sulfhydryl. This process gives rise to the synthesis of GSH from cysteine via the γ-glutamyl cycle, but would normally not be effective enough to replenish the supply of GSH [105]. Furthermore, in addition, lead inactivates δ-ALAD, GR, GPx, and GSH-S-transferase enzymes, and further depresses the levels of GSH [7,8]. Others are; SOD and CAT. Decreased concentration of SOD reduces the clearance of superoxide radical, while reduced CAT impairs the superoxide radical (O_2_^– •^) scavenging. Lead is also able to replace the zinc ions which serve as important co-factors for these antioxidant enzymes in trying to inactivate the enzymes [106], this is apart from targeting their sulfhydryl groups.

Lipid peroxidation is considered as a biomarker to oxidative stress and is one of the most investigated consequences of ROS on lipid membranes. The ROS takes electrons from the cell membranes and damages the cell by denaturing lipid that formed the membrane. Apart from lipid peroxidation, lead induces oxidation to Hb, resulting to hemolysis of the RBC. This happens due to ALAD inhibition and caused increased in the concentration of ALA substrate in blood, as well as urine. An elevated level of ALA, in turn, generates hydrogen peroxide and superoxide radical which goes to interact with oxy-Hb, resulting in the generation of hydroxyl radicals [21]. The aforementioned mechanism makes the cell highly vulnerable to oxidative stress and may also lead to cell death.

Bivalent cations such as Ca^2+^, Mg^2+^, Fe^2+^ and monovalent cations such as Na^+^, could also be substituted by Pb, thereby affecting various fundamental biological processes [107] in what is called as an ionic mechanism of lead action. Fundamental cellular processes such as intra and intercellular signaling, cell adhesion, protein folding and maturation, apoptosis, ionic transportation, enzyme regulation, release of neurotransmitters have been significantly affected by the aforementioned ionic mechanism [108]. This mechanism contributes principally to neurological deficits after lead replaces calcium ions; it becomes competent to cross the blood-brain barrier (BBB) at an appreciable rate. The lead then accumulates in astroglial cells (containing lead-binding proteins). The effects of lead toxicity are more pronounced in immature astroglial cells (developing nervous system) that lack lead-binding proteins. It (lead) easily damages the astroglial cells and obstructs the formation of myelin sheath, which is both involved in the development of BBB.

Key neurotransmitters such as protein kinase C can be affected by such lead replacement of calcium as picomolar concentration, protein kinase C regulates long-term neural excitation, and memory storage. Lead also affects the sodium ion concentration, which is responsible for enormous biological activities that are vital such as generation of action potentials in the excitatory tissues for the purpose of cell to cell communication, uptake of neurotransmitters (choline, dopamine, and GABA), and regulation of uptake and retention of calcium by synaptosomes. This interaction between lead and sodium seriously impairs the normal functioning of the aforementioned sodium-dependent processes [109].

### Possible treatment

Treatment may not be successful if the damage is too extensive particularly to the cells or tissue of the nervous system. Calcium disodium edentate (Ca-ethylenediaminetetraacetic acid [EDTA]) is given subcutaneous or intravenous for 3 days especially for cattle after lead exposure. A similar dose in dogs compartmentalized to 4 treatments/day given subcutaneously in combination with 5% dextrose for 2-5 days. If clinical signs persist, an extra 5-day treatment might be necessary after 1 week gap of stopping the therapy. Currently, there is no appropriate veterinary product that contains Ca-EDTA available commercially. Tissue deposition of lead is decreased by thiamine, which can also lessen the clinical manifestations. The most useful response seems to be resulted from combining thiamine and Ca-EDTA treatment [110]. The chelating agent succorer (meso 2, 3-dimercaptosuccinic acid [DMSA]) has been confirmed to be useful in birds and also efficient in dogs. DMSA has a much lesser side effects as compared to Ca-EDTA [111]. Cathartics such as a rumenotomy magnesium sulfate might be useful in removing lead from the GI tract. Tranquilizers or barbiturates could be used as a supportive therapy for cases manifested with convulsion episodes [110]. Antioxidant treatment combined with chelating agent might reduce the oxidative damage related to severe lead poisoning. However, antioxidants like N-acetylcysteine have been used in combination with DMSA [111]. In chelonian, it is possible to remove the ingested lead fragments from the stomach using endoscopically-guided forceps. This should be followed with 2 weeks therapy with Ca-EDTA [110].

## Conclusion

Lead poisoning has been known to humankind since centuries ago and became apparent during the industrial revolutions in the 18^th^ century. Lead has no recognized function biologically in the body, and thus when it enters the body, it causes serious health effects which might be permanent and lead to fatality.

## Authors’ Contributions

MAA and MNMH wrote and edited the manuscript according to the title. AWH, MYMS and MAR contributed the references for the content and edited some portions in this manuscript. All authors read and approved the final manuscript.
